# A Single Coronary Artery From the Left Coronary Sinus: The Continuing Conundrum

**DOI:** 10.7759/cureus.45844

**Published:** 2023-09-24

**Authors:** Dibyasundar Mahanta, Deepak Kumar Parhi, Shilpa Vinayak Gadade, Debasish Das

**Affiliations:** 1 Department of Cardiology, SUM Hospital, Bhubaneswar, IND; 2 Department of Cardiology, All India Institute of Medical Sciences, Bhubaneswar, Bhubaneswar, IND

**Keywords:** conundrum, left coronary sinus, artery, coronary, single

## Abstract

It is extremely rare to come across a single coronary artery during routine interventional cardiology practice. The incidence of single coronary arteries increases across congenital heart diseases. We report an extremely rare case of a single coronary artery arising from the left coronary sinus in an octogenarian presenting with anterior wall non-ST elevated myocardial infarction (NSTEMI) secondary to atherosclerotic occlusion of the proximal left main coronary artery (LMCA). It is often difficult to selectively engage a single coronary artery due to anomalous origin from the sinus; nonselective coronary sinus injection often suffices in visualizing the single coronary trunk dividing into left and right coronary arteries besides demonstrating the associated route and atherosclerotic anomalies.

## Introduction

Normal coronary blood supply includes the origin of the left main coronary artery (LMCA) from the left coronary sinus, which further bifurcates into the left anterior descending coronary artery (LAD) and the left circumflex artery (LCX) to supply the left side of the heart and the right coronary artery arises from the right coronary sinus to supply the right side of the heart. The anomalous single coronary artery is a rare congenital anomaly reported in 0.024 to 1% of cases across different series [1-3]. In a single coronary artery, a single coronary trunk arises from the coronary sinus which divides into the left and right coronary system and supplies the respective myocardium. A single coronary artery arising from the left coronary sinus is another rare coronary anomaly reported to be in the range of 0.1% to 0.9% across different series [4,5]. The incidence of single coronary arteries increases across complex congenital cyanotic heart diseases like pulmonary atresia, tetralogy of Fallot, and truncus arteriosus [6]. We report a rare case of a single coronary artery arising from the left coronary sinus in an 82-year-old female presenting with anterior wall non-ST elevated myocardial infarction (NSTEMI). A single coronary artery in an 82-year-old female with an atherosclerotic coronary artery is an extremely rare coronary anomaly, as most single coronary arteries present in the young or middle aged due to the anomalous course being susceptible to coronary compression.

## Case presentation

**Figure 1:**
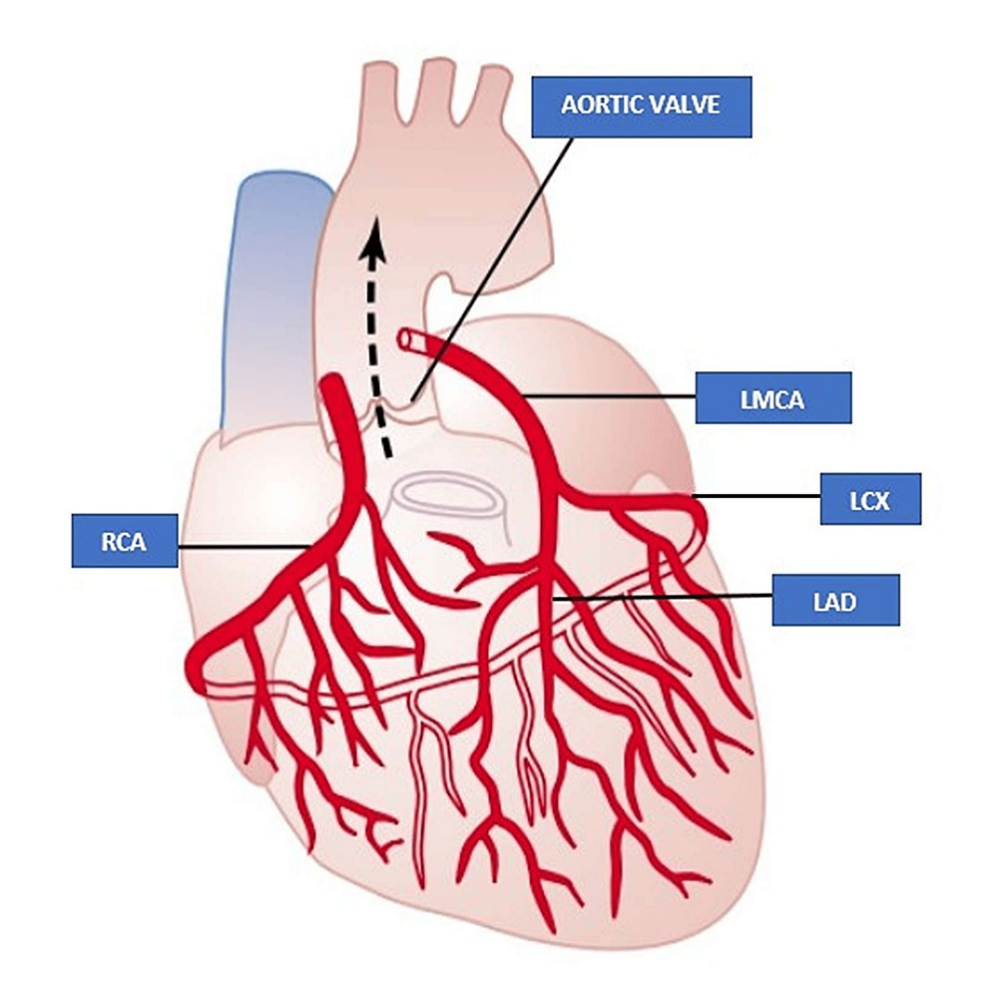
Normal coronary circulation Author's own creation LMCA: Left main coronary artery, LAD: Left anterior descending coronary artery, LCX: Left circumflex coronary artery, RCA: Right coronary artery

**Figure 2:**
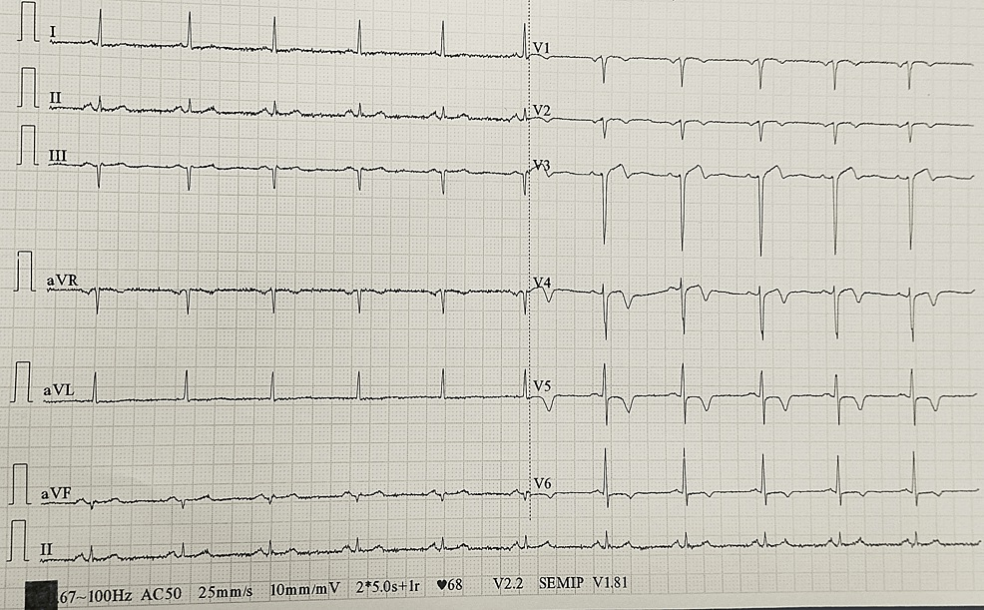
ECG showing T wave inversion in anterior precordial leads

**Figure 3:**
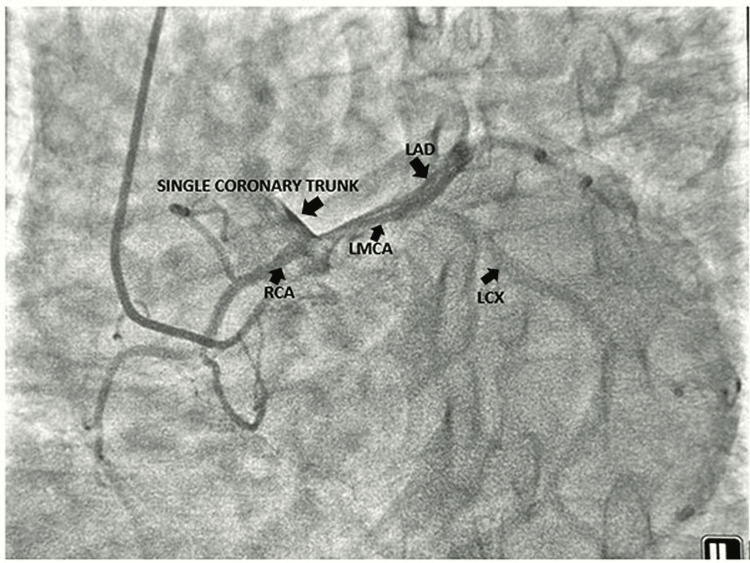
Single coronary trunk arising from the left coronary sinus bifurcating into the left main coronary artery and right coronary artery LMCA: Left main coronary artery, LAD: Left anterior descending coronary artery, LCX: Left circumflex coronary artery, RCA: Right coronary artery

Figure 1 depicts the normal coronary circulation. In our case, an 82-year-old female presented to the emergency department with retrosternal chest discomfort, diaphoresis, and palpitation for the last eight hours. She was diabetic and hypertensive and had been addicted to tobacco in the form of chewing betel nuts since a young age. She had effort intolerance New York Heart Association (NYHA) class II for the last three months. She had no history of presyncope or syncope. Her heart rate was 124 beats per minute and her blood pressure was 138/86 mmHg in the right arm supine position. She had no rest dyspnea and her baseline oxygen saturation was normal. Baseline EKG revealed symmetric T wave inversion in anterolateral precordial leads (Figure 2). Her echocardiography revealed no regional wall motion abnormality with normal left ventricular ejection fraction and degenerative aortic sclerosis. Troponin I was elevated (200 ng/L). She had a slightly elevated post-prandial blood sugar of 216 mg/dl and serum creatinine of 1.8 mg/dl. She was rushed to the cath lab for delineation of the coronary anatomy after initial stabilization with dual antiplatelet, high-dose statin, beta-blocker, nitrate, and low molecular weight heparin (LMWH). The left coronary artery could not be selectively engaged due to anomalous origin. Nonselective injection of the left coronary sinus with 10 ml of contrast with a tiger catheter revealed the origin of a single coronary artery from the left coronary sinus, which was further divided into the right and left coronary arteries supplying the respective territories (Figure 3). The proximal and mid part of the left main coronary artery revealed mild atherosclerotic luminal obstruction (20-30%) without much calcification. She was conservatively managed with aspirin, ticagrelor, atorvastatin, metoprolol, nitroglycerine, and LMWH for five days in view of acute coronary syndrome. She was discharged in hemodynamically stable condition after five days. Our case is an extremely rare illustration of an atherosclerotic single coronary artery arising from the left coronary sinus in an octogenarian. CT coronary angiography to delineate the anomalous course of a single coronary artery could not be done due to the presence of nephropathy.

## Discussion

Lipton et al. initially classified the single coronary artery that can arise from the right or left coronary sinus as follows: in the first category, the artery follows the course of either the left or right coronary artery, the second category of single coronary artery includes the aberrant course, including an anomalous artery running anterior to the pulmonary trunk, in between the pulmonary trunk and the aorta, and the anomalous artery running behind the aorta, and the third category includes the rarest variant with an absent left main coronary artery with the direct origin of the left anterior descending coronary artery and circumflex from the common trunk [7]. A single coronary artery traversing between the aorta and pulmonary artery carries the most malignant course with the risk of having ventricular tachycardia, myocardial infarction, and sudden cardiac death [8]. Most single coronary arteries are incidental findings during routine coronary angiography, and most of the patients are asymptomatic [9]. Younger patients (<30 years of age) suffer more sudden cardiac death either during rest or exertion secondary to systolic compression of the single coronary artery between the aorta and pulmonary artery. Although routine coronary angiography is the gold standard in delineating the single anomalous coronary artery, coronary CT angiography better delineates the three-dimensional anatomy of the anomalous single coronary artery, especially for looking at the variant that traverses between the aorta and pulmonary artery at the cost of the higher dose of radiocontrast exposure (80-100 ml). In persons unwilling to have radiation exposure, cardiac MRI is also a well-validated tool to delineate the three-dimensional anatomy and associated abnormalities in the case of a single coronary artery. A single coronary artery with a slit-like ostium, acute takeoff, and proximal intramural course is prone to suffer from obligatory coronary ischemia [10]. Although coronaries are usually free of atherosclerosis in single coronary arteries, they can have significant stenotic lesions, and successful coronary interventions, including coronary angioplasty and coronary artery bypass surgery (CABG) in single coronary arteries, have been reported [11]. Our case had a non-critical left main coronary lesion, which was conservatively managed. It is often difficult to engage a single coronary artery during the coronary intervention [12] and adequate knowledge about the anomalous origin and hardwires can overcome the difficulty during selective coronary cannulation. Our case is an extremely rare illustration of an atherosclerotic single coronary artery arising from the left coronary sinus in an octogenarian following the normal course of coronary arteries. Although rare to encounter, one may come across a single coronary artery even in extremes of age.

## Conclusions

We report an extremely rare case of a single atherosclerotic coronary artery arising from the left coronary sinus in an octogenarian presenting with non-ST elevation myocardial infarction (NSTEMI). It is often difficult to engage the anomalous origin of a single coronary artery from the left coronary sinus; nonselective injection of the left coronary sinus with 10-20 ml of contrast can visualize the same during difficult selective intubation. Although single coronary arteries in the young are commonly nonatherosclerotic, our case is a rare case of the atherosclerotic single coronary artery when it pertains to the elderly population.
